# MIIB: A Metric to Identify Top Influential Bloggers in a Community

**DOI:** 10.1371/journal.pone.0138359

**Published:** 2015-09-28

**Authors:** Hikmat Ullah Khan, Ali Daud, Tahir Afzal Malik

**Affiliations:** 1 Department of Computer Science and Software Engineering, International Islamic University, Islamabad, Pakistan; 2 Department of Management Information Systems, Ibn Rushd College for Management Sciences, Abha, Kingdom of Saudi Arabia; Semmelweis University, HUNGARY

## Abstract

Social networking has revolutionized the use of conventional web and has converted World Wide Web into the social web as users can generate their own content. This change has been possible due to social web platforms like forums, wikis, and blogs. Blogs are more commonly being used as a form of virtual communication to express an opinion about an event, product or experience and can reach a large audience. Users can influence others to buy a product, have certain political or social views, etc. Therefore, identifying the most influential bloggers has become very significant as this can help us in the fields of commerce, advertisement and product knowledge searching. Existing approaches consider some basic features, but lack to consider some other features like the importance of the blog on which the post has been created. This paper presents a new metric, MIIB (Metric for Identification of Influential Bloggers), based on various features of bloggers’ productivity and popularity. Productivity refers to bloggers’ blogging activity and popularity measures bloggers’ influence in the blogging community. The novel module of BlogRank depicts the importance of blog sites where bloggers create their posts. The MIIB has been evaluated against the standard model and existing metrics for finding the influential bloggers using dataset from the real-world blogosphere. The obtained results confirm that the MIIB is able to find the most influential bloggers in a more effective manner.

## Introduction

The concept of users being capable of generating content and being able to have social interaction has transformed the World Wide Web into the social web. The social web provides an opportunity to do social activities like interaction and participation at global level, forming virtual communities’ also known as social networks. These virtual communities allow users to share their views, ideas, knowledge, opinions, and even media-contents. The examples of such virtual communities include forums, web logs and wikis. A web log, usually known as a blog, enables users to express their views, experiences and opinions about certain topics. The topics are initiated by starting new posts which may contain text, image, media content and hyperlinks to other posts or web pages. The collection of blogs on the internet is known as the blogosphere. The Social interaction feature has motivated the researchers to include social concepts in their approaches to understand human behavior in a better and indirect manner.

In the physical world, the majority of people (83%) consults their family, friends or an expert over traditional advertising before going to any new restaurant, 71% of people act similarly before visiting a place or buying a prescription drug, and 61% of people do the same before watching a movie. In short, before make decisions, they talk, and they listen to other’s experience, views, and recommendations. The individuals whose views, opinions, and recommendations are required are termed in the relevant literature as the influentials [1].

The identification of influential bloggers in online communities and blogs is very significant. In technical blogs, the main goal is to discover quality content usually provided by an expert, whereas in marketing blogs, we primarily focus on identifying trustworthy customers. The companies can seek to find influential bloggers who can become some unannounced representatives for product uplift and marketing.

The current exponential growth of social web use has motivated researchers on addressing issues related to blogosphere [2]. Earlier research works related to the identification of influential bloggers were based on locating influential blog sites [3] and the study of the spread of influence among blog sites [4], [5], [6]. To identify influential bloggers, PageRank [7] and other ranking algorithms have been used to rank authors in academic social network [8]. PageRank has been adapted in [9] to rank the blogs sites, where the authors stated that the sparseness of the blog graph renders the traditional Web retrieval models inappropriate for the Blogosphere. A lot of research work has been done to find the influential users in the Blogosphere and are discussed later in the related work section.

In this paper, we propose a new metric named MIIB (Metric for Identification of Influential Bloggers) based on novel features and we compare it against the standard model as a baseline [10] and existing metrics [11]. The contributions of the proposed approach can be summarized as follows:

1. We propose five features. The novel features include the importance of blog where the bloggers submit their posts, a bloggers’ ability to remain active in the blog and also their ability to post on a consistent basis, the average length of the comments has been taken as a measure of eloquence.
2. We apply weights to each feature, according to its importance.
3. We are pioneer to propose the modular approach for a metric as the metric consists of three modules of Productivity, Popularity and BlogRank.
4. Individual features based analysis has been shown to depict how each feature contributes in the identification of influential bloggers which helps in the overall evaluation.
5. MIIB has been evaluated against the baseline of iFinder model [10] and metrics [11] and all three modules of Productivity, Populairty and BlogRank have also been evaluated to show their significance.
6. Evaluation has been performed using the standard ranking performance measures of Osim, Kendall Rank-Order Correlation and Spearman Rank Correlation.

The rest of the paper has been organized as follows: Section 2 introduces the related research works, section 3 provides the problem formulation and problem, section 4 introduces MIIB and its modules, section 5 provides information about experimental setup, the dataset used and the performance evaluation measures. In section 6, we discuss the results and evaluation of the individual features, modules and MIIB metric against the baseline model. Finally the paper is concluded in Section 7.

## Related Work

The domain of influential bloggers identification has been introduced in [12], where the basic model known as the influence flow model has been proposed. The model is based on the idea that active users can be influential. This model initially takes into consideration the features related to the bloggers and their posts. Then, it introduces a comprehensive model which is based on four features which include Recognition (based on how many comments received), Activity Generation (based on the number of comments posted), Novelty (based on inverse proportion of outgoing links) and Eloquence (length of comments) [10]. It includes a limited number of features and targets to find the bloggers who are influential based on the number of comments received by their posts and then compares with active users who have the most number of posts. It fails to consider the bloggers consistency and the importance of the blog site in which the bloggers post their content. The evaluation of the model has been done by comparison against PageRank, while it has been stated that such algorithms are not recommended for the domain of the Blogosphere.

Two other metrics to identify influential bloggers were proposed in [11]. These metrics known as MEIBI and MEIBIX, investigate the temporal aspect of the blogger’s activity and support time-aware identification of the influential bloggers. However, they take into consideration only a few features. The same work was further extended to propose two more metrics, BP-index and BI-index [13]. The former evaluates the productivity of the bloggers while the latter calculates the influence index of the bloggers. Then, the study includes an analysis of them separately as well as in combination. In all the four metrics, no new features were included even the less number of features were included in the new metrics. Also, all the indexes were based on H-index which is primarily used for ranking academic scholars and have its own limitations [14]. One of the main limitations is that it does not include all the comments and inlinks and also the H-top values become insignificant and we can have same H-index for two authors who have different number of comments and inlinks in total.

A recent model introduces two new factors of uniqueness and FacebookCount [15]. It also considers the sentiment of the content of the blog. It argues that the model can be further extended to include Twitter Share, G+1 etc. Another recent work presents the ranking model for blogs by introducing quality and temporal features [16]. It does not focus on the identification of influential bloggers, but considers the importance of blogger as an important measure. Another work ranks the top users using the topic into consideration and introduces a new measure of Osim as well [17].

A blog ranking metric, BI-Impact, has been proposed to identify influential blogs in a blogosphere [18]. The metric considers various factors such as the bloggers’ activity, interaction of a post and post content to compute the overall impact of the blog. Various weights have been proposed. Social network structure of the blogosphere has been exploited to find influential bloggers using the six network centrality measures [19]. They apply a centrality aggregation approach to compute the influence score of bloggers. Taking social network into consideration, another model, Longitudinal User Centered Influence (LUCI) [20], uses the interaction among bloggers and categorized them into four classes of introvert leaders, extrovert leaders, followers and neutrals. The higher classification accuracy results (90.3%) show the importance of the characteristics considered by LUCI. A recent work [21] proposes a method based on comments receive on each post and then compares the results with iFinder [10], which is our baseline as well. The authors conclude that the comments are more important than incoming links and iFinder gives too much importance to the inlinks. The results are similar to our findings as discussed later in the paper.

Motivated by the weaknesses in the existing literature in the domain of identification of influential bloggers, we propose a new metric that introduces more new features into the existing models. The model proposed in [10] has been taken as a baseline and then it has been further extended by the introduction of new modules which consists of previous and new features and the concepts of weights for features. The MIIB decomposes the main metric into different features so that their influence scores on overall influence be can be computed. We have also used for the first time the evaluation measures to compute the overlapping similarity, correlation and also the strength of the ranking results of MIIB and baseline methods.

## Problem Formulation and Problem Statement

In this section, we formulate and state our problem.

### Problem Formulation

In a blog, a topic is initiated by a blogger and the users can post their comment in it. The content is the post which may consist of text and links to other blogs. A blog post which draws the attention of other users is known as an influential blog post. The word attention here means that the blog post inspires other users to comment or create a link to blog posts. An influential blogger is the one who initiates the influential blog posts. The task is to find the top influential bloggers based on certain features which are related to bloggers, such as the ability to create new blogs, and blogs such as how many posts are there, how many users post their content etc. The weights assigned to the features depict the significance of the features. The topics discussed in the blog and the semantics of the content are out of this paper scope and have been left for the future work.

### Problem Statement

Given a set *B* of *N* bloggers, {*b*_1_, *b*_2_, …, *b*_*N*_} the problem of finding the influential bloggers can formally be defined as determining an ordered subset I of *K* bloggers,ordered according to their influence scores, S_*infl*_, such that *I* ⊆ *B* and *K* ≤ *N*, i.e., S_infl_(*b*_*j*1_) ≥ S_*infl*_(*b*_*j*2_) ≥, …, S_*infl*_(*b*_*jk*_). The set *I* contains the *K* most influential bloggers.

## The Proposed Metric

Initially, the features are discussed, and then the modules and the proposed model, MIIB, are presented. All the symbols used in the paper are recorded in Table 1 as follows:

**Table 1:** List of Symbols used in the paper.

Symbol	Remarks
*B*	Set of Bloggers
*P*	Set of Blog Posts
*S*	Set of blog Sites
*b*	*b* ∈ *B*
*p*	*p* ∈ *P*
*s*	*s* ∈ *S*
$N_{p}^{b}$	Number of blog posts posted by a blogger
$N_{d}^{b}$	Number of days blog posts posted by a blogger
${\text{S}}_{r}^{b}$	Score of regular posting of a blogger
$N_{l}^{b}$	Length of blog posts posted by a blogger
${\text{S}}_{a}^{b}$	Score of Average length of the blog posts posted by a blogger
$N_{c}^{b}$	Number of comments received on blog posts posted by a blogger
$N_{I}^{b}$	Number of Inlinks received on blog posts posted on a blogger
$N_{o}^{b}$	Number of outlinks in blog posts posted by a blogger
$N_{b}^{s}$	Number of Bloggers b who post in a blog site *s*
$N_{p}^{s}$	Number of posts posted in a blog site *s*
$N_{I}^{s}$	Number of in-links received by posts in a blog site *s*
$N_{c}^{s}$	Number of comments received by posts in a blog site *s*
${\text{S}}_{prod}^{b}$	Computed Score of Blogger *b* based on the productivity features
${\text{S}}_{popu}^{b}$	Computed Score of Blogger *b* based on the popularity features
${\text{S}}_{BRank}^{b}$	Computed Score of Blogger *b* based on the Blog site Rank features
${\text{S}}_{BRank}^{s}$	Computed Score of Weblog site *s* based on the Blog site Rank features
${\text{S}}_{infl}^{b}$	Final Influence Score of Blogger *b* based on all the features

### Factors Measuring the Blogger’s Influence

Generally, there are many factors that can be considered as a source of influence in the blogosphere. The baseline model proposed four features (number of posts, inlinks, comments and outlinks) and then proved their significance. The list of all the features, adopted or proposed, as follows:

**Activity (f1):** A Blogger’s ability to contribute in the blogosphere is an important feature so the number of blogs initiated by a blogger is the main contribution of a blogger. It is represented by $N_{p}^{b}$. This feature has been taken in about all the existing related works [10,11,13,15,18].
**Activeness (f2):** A blogger should remain active in a blog to be influential. It is possible that a blogger have submitted too many posts in a short period of time and remain inactive for the major part of period. An active blogger positively influences the ranking score of a post [18]. Activeness calculates the total number of days a blogger remains active in a blog. It is denoted by $N_{d}^{b}$.
**Consistency (f3):** A blogger should be consistent in his posting behavior to be taken as influential in the community. Consistency is the measure that blogger has posted blogs on regular basis. It has been argued [18] that bloggers should be consistent so that their impact should not vanish with time. It is a temporal feature and we find various existing works [11,13,15,16] takes time as an important feature. It calculates the period between the consecutive posts is considered. It has been denoted by ${\text{S}}_{r}^{b}$, and has been calculated by dividing the number of posts by the duration period of posting which has been calculated by subtracting the last posting date from first posting date. The score has been computed month-wise. The consistency is calculated using Eq 1, as follows:
  Consistency= Npb(max(postdate)-min(postdate)/30) (1)
**Recognition (f4):** The number of comments received by the posts of a blogger shows the recognition of the blogger in the community. It has been represented by $N_{c}^{b}$.
**Authority(f5):** In web based ranking algorithms [7], the incoming hyperlinks denote authority and it has been argued that it is more important to have inlinks from another blog than receiving comment on blogs [13]. The number of inlinks received on posts of blogger denotes their authority and has been Represented by $N_{I}^{b}$.
**Novelty (f6):** The number of outlinks depicts the lesser novelty of a blog, but in recent indexes, it has been argued that outlinks are important and should not be given less weightage. It has been dented by $N_{o}^{b}$. As this is an inverse measure, so in individual features, top results include those bloggers who have the most number of posts but less number of outlinks. Merely considering the less number of outlinks then those bloggers are returned who have no posts or very less number of posts and considering that the results would be meaning-less.
**BlogRank (f7):** BlogRank is based on the assumption that for a blogger to be influential, he/she should be posting on top blog sites. This feature first computes the important blogs and then the blogger who posts at higher ranking blogs should be regarded as more influential. It has been denoted by ${\text{S}}_{BRank}^{s}$.
**PostLength (f8):** The length of the post has been regarded as measure to show the eloquence of the blogger. The feature, denoted by the symbol $N_{l}^{b}$,represents the sum of characters of posts posted by the blogger *b*.
**NormalizedPostLength (f9):** It can be argued that sometimes blogger may post too lengthy content that can give him very high score, we here introduce the normalized comment as additional measure of influence. The feature, denoted as ${\text{S}}_{a}^{b}$, is calculated by dividing the sum of length of posts of the blogger *b* by the number of posts by the blogger.

The list of features and their objectives are given in Table 2 as follows:

**Table 2:** List of Features and their purpose.

Feature N0	Feature title	Remarks
f1	Activity	To measure the post initiating capability of the blogger
f2	Activeness	To measure the blogger ability to remain active in the blog
f3	Consistency	To measure the consistent posting behavior of the blogger
f4	Recognition	To measure how much other bloggers recognize the blogger
f5	Authority	To measure how much authority is given in the blog to the blogger
f6	Novelty	To measure how much novel content is posted by the blogger
f7	BlogRank	To measure the significance of blog in which blogger post
f8	PostLength	To measure the eloquence of the content posted by the blogger
f9	NormalizedPostLength	To measure the normalized quality of content posted by the blogger

### The Modules of MIIB

MIIB consists of three modules of productivity, popularity and BlogRank. The score of each module is calculated separately and each feature is given a certain weight. The modules are now briefly described.

#### Productivity Score

A blogger is considered productive and influential if he/she initiates new blogs consistently and regularly. The productivity score has been calculated using the activity, consistency, and activeness features. Activity is a blogger’s ability to create new posts which is the main important characteristic [10,11,13] while the remaining characteristics depend on it. The baseline model [10] takes into consideration only the length of comments as the eloquence measure to find influential bloggers. It can be argued that the total number of comments is not a good measure as few comments may consists of too much lengthy content, so NormalizedPostLength has been introduced which calculates average comment length. The Influence score based on Productivity can be computed using the Eq 2:

$$S_{Prod}^{b}=w_{p}N_{p}^{b}+\left(w_{d}N_{d}^{b}+w_{r}S_{r}^{b}\right)+\left(w_{l}N_{l}^{b}+w_{a}S_{a}^{b}\right)$$ (2)

Where *w*_*p*_ is the weight of blogger activity, *w*_*d*_ and *w*_*r*_ are the weights of activeness and consistency respectively and *w*_*l*_ and *w*_*a*_ are the weights of PostLength and normalizedPostLength respectively. The weight of activity is 2 as it is the most important characteristics to measure the productivity of the blogger, while the remaining features depend on activity so they have been given the weights of 0.5 so that the combined effect of each part should be 1 and thus overall all remaining four feature have been given same weight of 2 as that of activity.

#### Popularity Score

Popularity refers to the importance that has been given to the blogger within the community by the other virtual community members in the forms of comments and inlinks. It can be argued that a comment can be positive or negative in its feedback towards the blog, but inlinks show the direct influence and depicts authority of the blogger within the community. Outlinks is the reversely proportional to the novelty and this has been subtracted from the recognition part. The influence score is calculated using Eq 3, given as follows:

$$S_{popu}^{b}=\;w_{c}N_{c}^{b}+\left(w_{I}N_{I}^{b}-w_{o}N_{o}^{b}\right)$$ (3)

where ***W***_***c***_, ***W***_***I***_ and ***W***_***O***_ represents the weights of comments, inlinks and outlinks respectively and having the values of 1,2 and 1 respectively, which suggest the more importance is given to the inlinks than comments. The inlink feature has been given more weight and importance in the existing works [11,13,18]. In addition, the statistics given in Table 3 validate the importance of inlinks over comments in blog posts.

**Table 3:** TUAW Dataset Statistics.

Bloggers	51
Posts	17,831
Inlinks	53,575
Comments	2,67,949
Weblogs	6,655
Inlinks per post	3.004
Comments per post	15.027
Posts per Blogger	3496.6
Average Post Length	1321.225

#### Blog Quality Score

It is proposed that the importance of blog where the bloggers post is a significant feature. MIIB introduced the inclusion of the top blog as quality measure and thus the influence score of bloggers has been computed using equation can be computed using Eq 4 given as follows:

$$S_{BRank_{i}}^{s}=\;({\text{N}}_{b_{i}}^{s}+\;{\text{N}}_{p_{i}}^{s}+\;{\text{N}}_{I_{i}}^{s}+\;{\text{N}}_{C_{i}}^{s})$$ (4)

Where $S_{BRank}^{s}$ represents the web site rank calculated using the four features added together. Then, the top bloggers have been computed who have the most number of blog posts on the top weblogs and the score has been represented as $S_{BRank}^{b}$.

#### The Influence Score

Finally, the influence score, SInflb, of the blogger is based on all the features has been calculated by the weighted accumulative sum of the three modules, using Eq 5, as follows:

$$S_{infl}^{b}=w_{prod}S_{prod}^{b}+w_{popu}S_{popu}^{b}+w_{bank}S_{Brank}^{b}$$ (5)

Where ***w***_***prod***_ is the weight of productivity module and has been given 0.4, ***w***_***popu***_ is the weight of popularity module and its values has been set 0.4 and ***w***_***bank***_ is the weight of the BlogRank module and the value has been set 0.2. Existing work [13] verify that both productivity and influence have a strong relationship. So we consider both the modules and assign the same weight. As the proposed module BlogRank is highly correlated to MIIB so it has been given less weight (0.2 only).

## Experimental Setup

Here we discuss the dataset used to evaluate MIIB metric and the performance evaluation measures that we have used.

### TUAW Dataset

Apple started its weblog, The Unofficial Apple Weblog (TUAW), to publish new stories which cover a variety of topics which includes providing help to users and targeted marketing. As a technology blog, TUAW used to provide opportunity to users to comment, give opinions and discuss about the topics of blogs posts. The blog has recently been shut down (refer to this link for more details: http://www.theverge.com/2015/1/30/7949485/aol-shutting-down-tuaw-apple). A dataset extracted from TUAW has been developed and used by the baseline model [10]. We have used the dataset used in [11] which provides computation of all the required attributes. The dataset is freely available for research (Download link: http://users.sch.gr/lakritid/code.php?c=2). In addition, it is a comparatively bigger dataset having blogs of five years from 2004 to 2008. The dataset statistics are given in Table 3.

### Performance Evaluation Measures

MIIB has been evaluated against the baseline model by using performance evaluation measures discussed as follows:

#### OSim

Osim is used to measure the overlapping similarity between two lists or results of two ranking methods [17]. It is calculated by computing the intersection of the two lists normalized by the number of records in consideration. In this work, we compare the results to analyze how many bloggers are common using various metrics, proposed methods and its modules.7. For two ranked lists A and B, Osim for top 10 results can be computed as follows:

OSim =(A U B)/k (6)

#### Spearman's Rank-Order Correlation

Spearman's rank order correlation is a technique to compute a correlation coefficient between the ranking orders of scores on two variables. In this case we will analyze the correlation between the results of the modules of the MIIB and also to compare the results of existing metrics and proposed method. Spearman correlation has been used to compare various metrics to find influential bloggers [11]. Spearman rank-order correlation, given as follows:

$$Spearman\;Rank\;Order\;Correlation=1-6{\sum^{\;}}^{\;}k\left(k^{2}\;-1\right)$$ (7)

Where d represents the differences of ranks between the two ranking orders and *n* is the number of items in each case. In our case, we are taking top 10 bloggers, so k is equal to 10.

#### Kendall's Rank Correlation

Kendall's rank correlation is a measure to determine the strength of dependence between two variables. It is a measure that considers how much variation lies between two different ranking results. The variation inn ranking helps to analyze the reasons of different ranks for bloggers using various metrics and models. It is represented by **τ** and calculated using the following formula:

τ= (number of concordant pair)-(number of dicordant)/((1/2)n(n-1)) (8)

## Results and Discussion

The evaluation consists of four steps. Firstly, the results of the top ten bloggers based on each feature have been shown which helps us to analyze the results of the baseline and MIIB in a better manner. Secondly, MIIB has been compared with the baseline model. Thirdly, the significance of each module has been discussed. Lastly, the standard ranking evaluation measures of OSim, Kendall and Pearson Rank-order correlation have been used to perform the evaluation.

### Feature-based Evaluation

Table 4 provides the list of the top ten bloggers based on single features. S.McNulty has been ranked at top position in four significant features (Activity, Activeness, comments, BlogRank) and no other blogger enjoys such high ranks in individual features. Now, if we search for the blogger who enjoys the top ranking in the most number of features, then we find Erica Sadun to be among top five ranks in about all the features. So both S.McNutty and E.Sadun can be anticipated as the candidates for top overall influential bloggers.

**Table 4:** List of top bloggers based on each single feature.

	F1-noofposts	F2-nooddays	F3-consistency	F4-com	F5-inlink	F6-outlink	F7-blogrank	F8-len	F9-avglength
1	Scott McNulty	Scott McNulty	Barb Dybwad	Scott McNulty	Cory Bohon	Brad Hill	Scott McNulty	Erica Sadun	Weblogs, Inc.
2	Dave Caolo	Dave Caolo	David Chartier	Erica Sadun	Erica Sadun	C.K. Sample, III	Erica Sadun	David Chartier	Chris Ullrich
3	David Chartier	David Chartier	Sean Bonner	Dave Caolo	Robert Palmer	Michael Sciannamea	Dave Caolo	Scott McNulty	Pariah S. Burke
4	Erica Sadun	Erica Sadun	C.K. Sample, III	David Chartier	Dave Caolo	Greg Scher	David Chartier	Dave Caolo	Jason Clarke
5	C.K. Sample, III	Michael Rose	Erica Sadun	Victor Agreda, Jr.	Mike Schramm	Dori Smith	Cory Bohon	Mat Lu	Christina Warren
6	Mat Lu	Mat Lu	Scott McNulty	Mat Lu	Michael Rose	David Touve	Victor Agreda,Jr	Michael Rose	Brett Terpstra
7	Laurie A. Duncan	Cory Bohon	Dave Caolo	Cory Bohon	Mat Lu	Marc Orchant	Mat Lu	C.K.Sample, III	Scott Granneman
8	Cory Bohon	Laurie A. Duncan	Robert Palmer	Michael Rose	Steven Sande	Damien Barrett	Michael Rose	Laurie A. Duncan	Joshua Ellis
9	Michael Rose	Mike Schramm	Mat Lu	Mike Schramm	Scott McNulty	Jan Kabili	Mike Schramm	Cory Bohon	Caryn Coleman

The comparison of D.Caolo and D.Chartier is also interesting as both are ranked in top five in many features based ranking, but none is ranked on top position in the feature-based results. D.Caolo is ranked relatively high in most of the features and should be ranked higher than D.Chartier. C.Bohon has been ranked top bloggers who get the most number of inlinks but he is not ranked in the top five rankings of any other feature. This sets up to compare MIIB metric with the standard baseline model.

**Fig 1:**
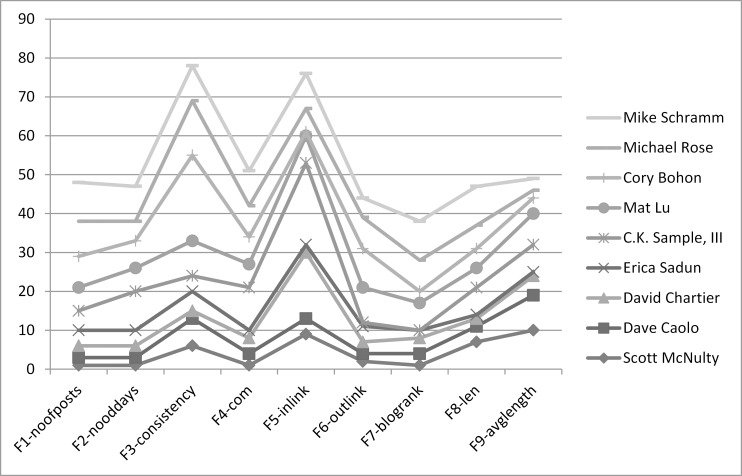
The top influential bloggers based on single features.

Fig 1 shows the rank variation of each blogger using each feature. If we analyse the bloggers ranking based on single features in chart as shown in Fig 1, it reveals that Scott McNulty enjoys higher ranks than C.K.Sample III who has more variations in the ranks. Comparing the ranking of Dave Caolo and David Chartier, both enjoy similar overall ranks, but differ a lot in case of inlinks, which is an important feature.

### Comparison of MIIB and the baseline

First of all, let us compare the cases of top influential bloggers ranked by both the baseline model and the MIIB respectively. S.McNutty has been ranked as top influential by MIIB however the baseline model does not rank him in top ten even. All the three modules productivity, popularity and quality have also ranked S.McNutty as top influential blogger as given in Table 5. This result is as predicted in feature wise analysis and depicts the flaws in the baseline model.

**Table 5:** A comparison of Top Results of modules, MIIB vs the baseline.

Rank	Productivity	Popularity	Quality	Baseline	MIIB
1	Scott McNulty	Scott McNulty	Scott McNulty	Cory Bohon	Scott McNulty
2	Dave Caolo	Erica Sadun	Erica Sadun	Robert Palmer	Erica Sadun
3	David Chartier	Dave Caolo	Dave Caolo	Mat Lu	Dave Caolo
4	Erica Sadun	Cory Bohon	David Chartier	Christina Warren	David Chartier
5	C.K. Sample, III	David Chartier	Cory Bohon	Dave Caolo	Cory Bohon
6	Mat Lu	Victor Agreda, Jr.	Victor Agreda, Jr.	Chris Ullrich	Victor Agreda, Jr.
7	Laurie A. Duncan	Mat Lu	Mat Lu	Steven Sande	Mat Lu
8	Cory Bohon	Michael Rose	Michael Rose	Michael Rose	Michael Rose
9	Michael Rose	Mike Schramm	Mike Schramm	Victor Agreda, Jr.	Mike Schramm
10	Mike Schramm	Robert Palmer	Robert Palmer	Jason Clarke	Robert Palmer

The baseline method ranks C. Bohon as the top influential blogger. Single feature wise analysis shows that he is 8^th^ in activity, 7^th^ in the activeness and does not appear in the top five positions in any of the features. Only exception is in regards to inlinks where he is top ranked blogger. So it depicts that the baseline gives too much importance to the inlinks feature while the MIIB gives importance to all the other features. C.Bohon does not enjoy high ranks in module based analysis as well. E.Sadun has been ranked high (second) as expected in the MIIB but she is not ranked in the baseline method. Also the modules of popularity and quality rank her highly. D.Caolo and D.Chartier have been ranked third and fourth respectively by the MIIB as expected, but the MIIB rank them significantly low. Considering the ranking of baseline, the top ranked C.Bohon has been ranked at 5^th^ position as it has been ranked in similar positions in single feature as well as at module levels which suggests that the MIIB provides more accurate and realistic results than the baseline. As anticipated in feature-wise discussion, E.Sadun has been ranked second by the MIIB but has not been ranked in top ten in the baseline results.

### Comparison of MIIB vs Existing Metrics

Let us consider the MIIB with the existing metrics of MIBI and MIBIX [11] with the help of results presented in Tables 4, 6 and 7. The high values of OSim given in Table 7 show that the overall results are similar which depicts that our results are valid. But the correlation results are low, which shows that the proposed metric provides different ranking orders.

**Table 6:** A comparison of Top results of MIIB vs Existing Metrics.

Rank	MIBI [11]	MIBIX [11]	MIIB
1	Cory Bohon	Cory Bohon	Scott McNulty
2	Robert Palmer	Robert Palmer	Erica Sadun
3	Steven Sande	Steven Sande	Dave Caolo
4	Erica Sadun	Erica Sadun	David Chartier
5	Michael Rose	Christina Warren	Cory Bohon
6	Mike Schramm	Michael Rose	Victor Agreda, Jr.
7	Christina Warren	Mike Schramm	Mat Lu
8	Dave Caolo	Mat Lu	Michael Rose
9	Mat Lu	Dave Caolo	Mike Schramm
10	Brett Terpstra	Brett Terpstra	Robert Palmer

**Table 7:** A comparison of MIIB vs Existing Metrics using Evaluation Measures.

	OSim	Spearman Correlation	Kendall Correlation
**MIBI vs MIBIX**	1	0.9515	0.8667
**MIBI vs MIIB**	0.8	0.2242	0.2
**MIBIX vs MIIB**	0.8	0.22	0.16

Let us discuss the cases of three top bloggers ranked by MIBI and MIBIX to compare with MIIB results. Both MIBI and MIBIX rank Cory Bohon as top blogger, while he is only top ranked in inlinks and does not enjoy rank among the top five positions in any other feature. So, MIIB properly ranks him 5^th^ in the list. In the case of Robert Palmer, who enjoys 8th in the consistency feature only, 3^rd^ in inlinks and does not have a rank in top ten in any other feature. Existing metrics rank him at 2^nd^ position while the MIIB rank him in 10^th^ position. The ranking of Steven Sande provides an even better comparison as he is ranked 8th in inlinks only and does not appear in top ranking of any other feature as evident from Table 4, but MIBI and MIBIX rank him at 3^rd^ position which seems improper. It is evident from the above discussion of three cases that MIBI and MIBIX gives too much importance to inlinks. It has also been argued [13] that an incoming link may be in favor or against a certain post so giving too much importance may not be a proper.

### Module-wise Evaluation

**Fig 2:**
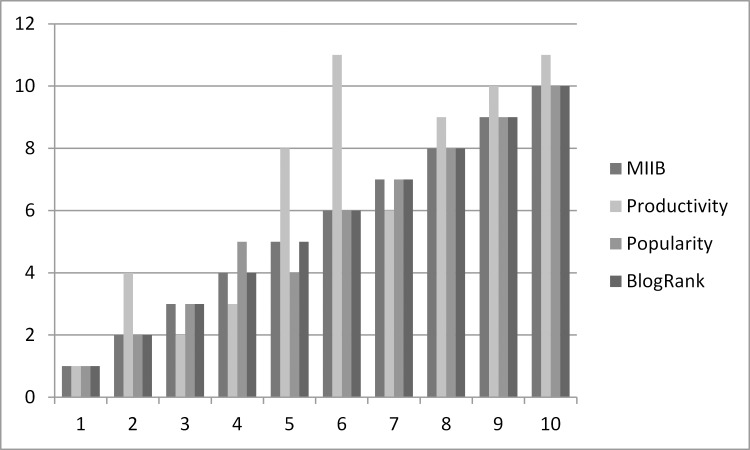
Module-wise Comparative Analysis.

**Fig 3:**
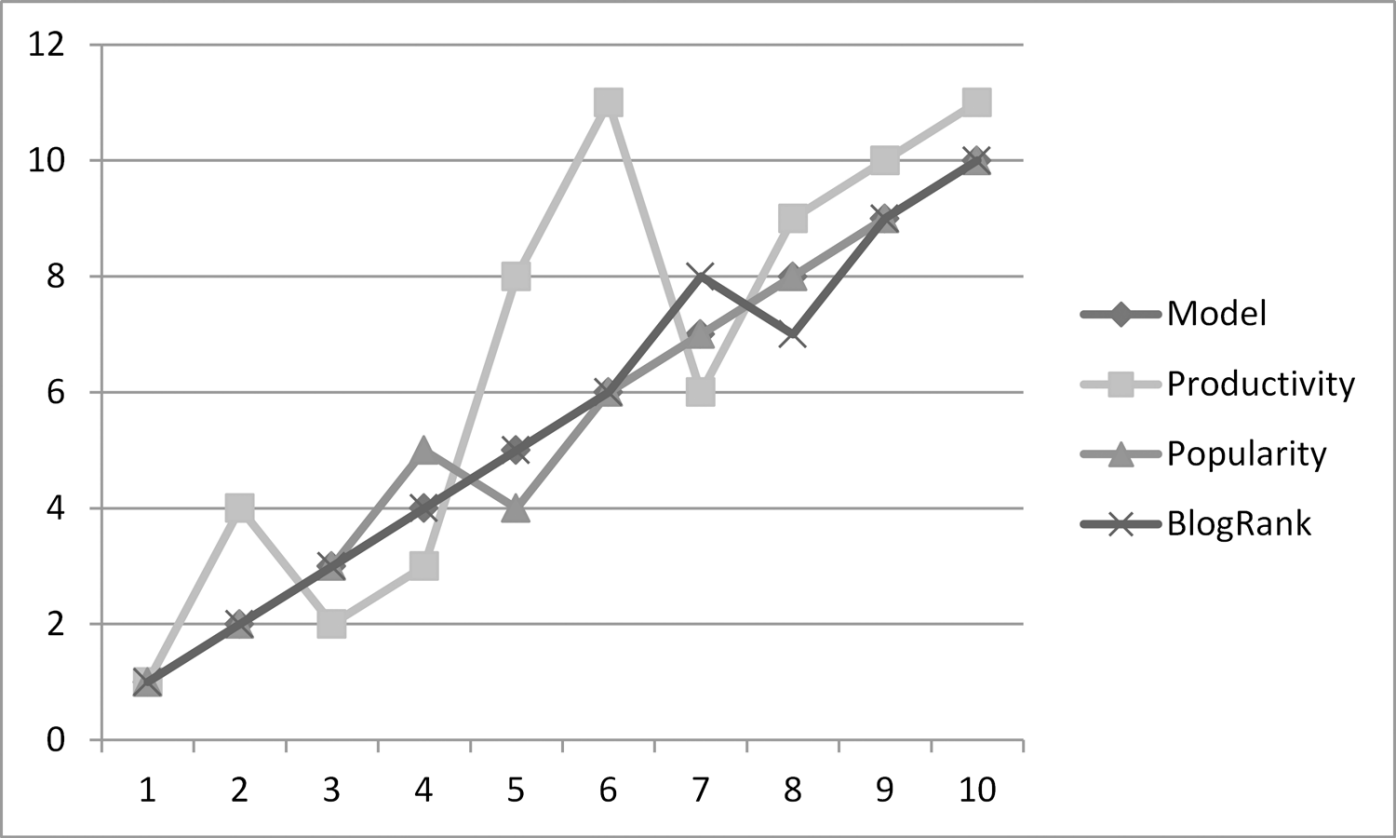
Comparative analysis of the Modules of MIIB to find Top Influential Bloggers.

The Fig 2 shows that comparison of results of the modules of the MIIB in finding the top influential bloggers in the blogosphere. The analysis reveals that overall ranking of bloggers in each module is consistent and no main divergence in top positions is found. MIIB is exactly in line with BlogRank and absolutely no difference is visible which supports our assumption that top influential bloggers post at top blogs. The popularity is another measure of direct influence and the top results of the MIIB are similar as results produced by module popularity. The only difference between the MIIB and the productivity module is visible, which again proves our point that merely initiating more number of posts is not the true measure of influence and is inaccurately given extra importance in existing models. The module-wise comparative results presented in line chart given in Fig 3.

This chart validates our above mentioned discussion and proves that all the modules depict their importance in finding influential bloggers.

### MIIB Metric Evaluation using Performance Evaluation Measures

It is another contribution that the results of modules and the MIIB have been evaluated using the performance evaluation measures, which have not been used in results evaluation of any of the existing models for finding the influential bloggers in the blogosphere. The results of each of the performance evaluation measures are discussed separately. The comparative analysis is based on top k i.e., 10, 20, 30 and for the entire dataset have been shown.

Pearson rank order correlation has been used to compute the correlation coefficient between the results of the modules of the MIIB and also between the modules and the MIIB. The results given in Table 8 reveal that BlogRank has the highest correlation as compared to other two modules. Popularity is more correlated to the MIIB as it has features that directly related to inference as compared to Productivity.

**Table 8:** Person Rank-order Correlation of the modules and the MIIB.

Comparison between	Dataset	Top 30	Top 20	Top 10
Productivity vs Popularity	0.8842	0.8641	0.8316	0.8362
Productivity vs BlogRank	0.8962	0.8784	0.8502	0.8625
Popularity vs BlogRank	0.9997	0.9996	0.9994	0.9988
Productivity vs MIIB	0.8962	0.8784	0.8502	0.8625
Popularity vs MIIB	0.9997	0.9996	0.9994	0.9988
BlogRank vs MIIB	1	1	1	1

Kendall correlation shows the strength of correlation the modules and the MIIB and also it considers the variations in the ranking order of the two approaches. It is also interesting to note from the Kendall results presented in Table 9 that similar results are observed as those of Pearson rank order correlation given in Table 9.

**Table 9:** Kendall Correlation of the modules and the MIIB.

Comparison between	Dataset	Top 30	Top 20	Top 10
Productivity vs Popularity	0.70732	0.61839	0.62105	0.64444
Productivity vs BlogRank	0.73415	0.65057	0.63158	0.68889
Popularity vs BlogRank	0.95406	0.96782	0.98947	0.95556
Productivity vs MIIB	0.73415	0.65057	0.63158	0.68889
Popularity vs MIIB	0.95406	0.96782	0.98947	0.95556
BlogRank vs MIIB	1	1	1	1

OSim, also known as, Overlapping similarity, measures the common resultant values of the two approaches. Table 10 contains the Osim results for different values of k i.e., the number of bloggers. It displays that how many resultant bloggers are common among different modules and the MIIB. It is understandable that for the entire dataset, this value will be 1. The proposed module, BlogRank, produce similar results as those of the MIIB which suggests the importance of the blogs where bloggers create their posts. All the three modules have similar values for top 30 bloggers, which signifies that all the modules are important and contribute to finding the top influential bloggers of the blogosphere.

**Table 10:** Osim of the modules and the MIIB.

Comparison between	Dataset	Top 30	Top 20	Top 10
Productivity vs Popularity	1	0.93333	0.8	0.8
Productivity vs BlogRank	1	0.93333	0.85	0.8
Popularity vs BlogRank	1	1	0.95	1
Productivity vs MIIB	1	0.93333	0.85	0.8
Popularity vs MIIB	1	1	0.95	1
BlogRank vs MIIB	1	1	1	1

## Conclusion

A novel weighted metric has been proposed to find influential users in the blogosphere based on nine features. The productivity and popularity of the individual bloggers have been computed based on features and it has been proven that it is important to consider the importance of the blog site where the bloggers share their posts. Feature-wise, module-wise and complete MIIB metric versus baseline methods evaluation have been performed with the help of standard performance evaluation measures using real world community of web bloggers and the obtained results confirm that the proposed methods identify the influential bloggers in a more effective manner. The model can further be used for any dataset where the more features and modules may be added and the new weights can be introduced.
